# Occlusion néonatale par diaphragme duodénale: à propos d’un cas

**DOI:** 10.11604/pamj.2016.25.85.9871

**Published:** 2016-10-17

**Authors:** Eric Mbuya Musapudi, Didier Tshibangu Mujinga, Guy Nday Ilunga

**Affiliations:** 1 Département de Chirurgie, Cliniques Universitaires de Lubumbashi, Faculté de Médecine, Université de Lubumbashi, Lubumbashi, République Démocratique du Congo

**Keywords:** Occlusion néonatale, diaphragme duodénal, prise en charge chirurgicale, Neonatal obstruction, duodenal diaphragm, surgical management

## Abstract

Le diaphragme duodénal est l’une de rare forme d’atrésie duodénale décrite, qui provoque l’occlusion intestinale pendant la période néonatale. La cause est embryologique. Les auteurs rapportent le cas d’un nouveau-né de 17 jours, hospitalisé et prise en charge aux cliniques universitaires de Lubumbashi pour occlusion duodénale et dont le constat per opératoire était un diaphragme duodénal. Son évolution était bonne après l’intervention chirurgicale.

## Abstract (en)

Duodenal diaphragm is one of the rare types of duodenal atresia described, causing intestinal obstruction in the neonatal period. It is caused by embryological factors. We report the case of a 17-day-old newborn hospitalized and treated for duodenal obstruction at the University clinics in Lubumbashi and whose intraoperative finding was duodenal diaphragm. Evolution was good after surgery.

## Introduction

L´atrésie duodénale est une embryopathie qui porte sur l´intestin crânial et qui entraîne une absence complète de la lumière duodénale. Elle est l´une des anomalies les plus courantes chez les nouveaux nés, et représente près de la moitié de tous les cas d´obstruction intestinale néonatale. Il s’agit d’une anomalie qui répond bien au traitement chirurgical [1, 2]. Des anomalies vasculaires, de migration des cellules nerveuses et un défaut de recanalisation de la lumière duodénale pourraient être à l´origine de l´atrésie, mais la cause exacte reste inconnue. Sa prévalence se situe entre 1 sur 6000 et 1 sur 10.000 naissances [1]. Huang [3] à Taiwan décrit 13 cas d’occlusion par diaphragme duodénal dans son étude menée pendant 10 ans dont 61,5% de cas diagnostiqués tardivement, alors que Chen [2] en chine décrit 55 cas de diaphragme duodénal sur une période de 9 ans.

Le diagnostic peut être posé avant la naissance dans 50% de cas devant une dilatation de l’estomac et du duodénum proximal visible à l’échographie fœtale réalisée au troisième trimestre. Le signe radiographique de l’atrésie duodénale est la «double bulle» avec distension gazeuse de l´estomac et du duodénum proximal et absence totale de gaz intestinaux distale [1]. La rareté de cette pathologie dans le monde motive cette publication. L’objectif est de décrire le tableau clinique et la prise en charge d’un cas de diaphragme duodénal observé aux cliniques universitaires de Lubumbashi en Mai 2015.

## Patient et observation

Il s’agissait d’un nouveau-né de sexe masculin de 17 jours de vie, amené en consultation dans le service de chirurgie des cliniques universitaires de Lubumbashi en date du 28 Mai 2015 pour vomissements postprandiaux depuis sa naissance avec notion d’émission de méconium après sa naissance.

Il a été d’abord traité dans un centre hospitalier de la place aux antibiotiques associant le cefotaxime et l’ampicilline et à la quinine sans succès. La survenue de la perte pondérale et la persistance de vomissements qui devenaient bilieux avaient motivé la famille à amener l’enfant aux cliniques universitaires pour une prise en charge adéquate.

**Figure 1:**
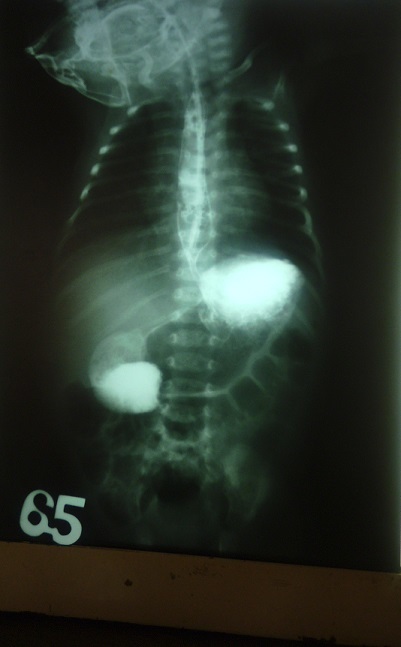
Transit oesogastro-duodénal à la gastrographine qui montre une dilatation du duodénum et un arrêt du transit est noté au niveau de D3

**Figure 2:**
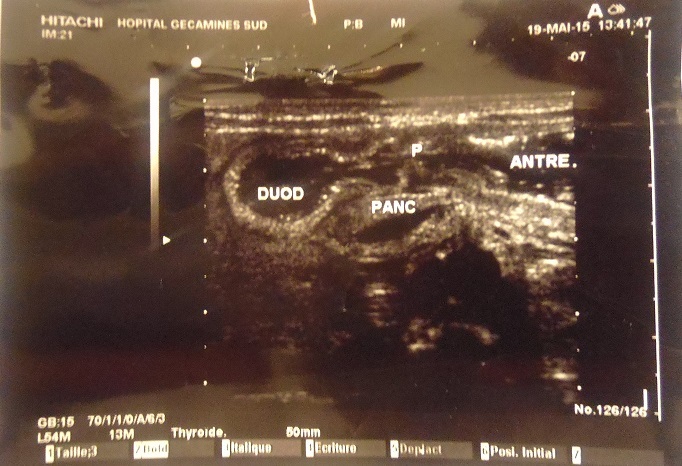
Ehographie abdominale qui montre rétention et dilatation du duodénum en regard de la tête du pancreas

A l’examen physique, les paramètres anthropométriques se présentaient comme suite: Poids: 3,300kg (poids de naissance, 3450 g), Taille: 47 cm, Périmètre crânien: 36 cm, Périmètres brachial et thoracique respectivement, 14 et 37cm. L’examen somatique sommaire avait montré un nouveau-né rosé, sans signes de déshydratation, les signes vitaux dans les normes physiologiques. Le thorax était symétrique, de bonne ampliation, mamelon surélevé, aréole mammaire de 10cm. L’examen des poumons et du cœur était normal Son abdomen n’était pas ballonné, ombilic bien cicatrisé, souple, dépressible, sans visceromegalie, le péristaltisme était présent. Les membres étaient eutrophiques et symétriques. Au toucher rectal, la marge anale était propre, le sphincter anal tonique, rectum vide, le cul-de-sac de douglas non bombant. L’examen neurologique sommaire avait mis en évidence un nouveau-né bien éveillé, tonique, le Moro bien exécuté, il agrippait fortement. La succion et la déglutition étaient vigoureuses. Les examens paracliniques qui ont été demandés et réalisés avaient montré : La radiographie avec transit à la gastrograhine n’avait objectivé ni d’obstacle œsophagien, ni de hernie hiatale. L’estomac était d’aspect normal. Cependant, une dilatation duodénum et un arrêt du transit étaient noté au niveau de D3 (Figure 1). La radiographie de l’abdomen sans préparation, n’avait pas mis en évidence des signes du syndrome occlusif. L’échographie avait montré un passage libre au niveau pylorique. Une importante rétention et dilatation du duodénum en regard de la tête du pancréas suggérant un diagnostic du pancréas annulaire ou d’atrésie duodénale (Figure 2).

Les diagnostics différentiels étaient, l’atrésie duodénale et le pancréas annulaires et une laparotomie exploratrice avait été décidée. Le bilan sanguin préopératoire a montré, un groupe sanguin A rhésus positif, Temps de Saignement: 1 minute et 00seconde, Temps de Coagulation : 3minutes et 30secondes, Hb: 20,7g%, Hct: 61%.

Apres une visite pré-anesthésique et une réanimation préopératoire, il a été opéré un jour après son admission.

**Figure 3:**
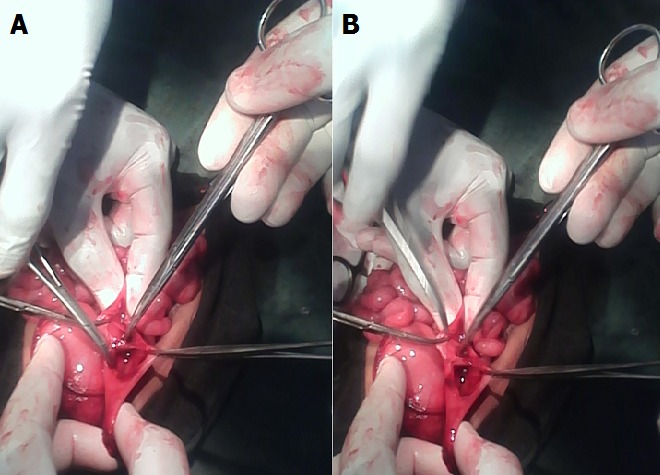
(A,B) exposition per opératoire de la membrane endoluminale après une incision longitudinale réalisée au niveau de la sténose

L’ouverture de la cavité abdominale avait montré, l’estomac fortement dilaté ainsi que les deux premières portions duodénales, le reste des anses était rétréci et ratatiné. L’ouverture de l’arrière cavité des épiploons au travers le facia placide visualisait un pancréas normal écartant ainsi le diagnostic d’un pancréas annulaire. Continuant l’exploration, une zone d’affaissement au niveau de la troisième portion du duodénum avec dilatation en amont a été mise en évidence. L’inexistence de brides extrinsèques enserrant le duodénum au niveau de l’affaissement, a permis d’évoquer un obstacle intraluminal. L’incision longitudinale à cheval entre la portion dilatée et la portion rétrécie avait mis en évidence un diaphragme sous forme d’une membrane endoluminale obstruant la lumière intestinale (Figure 3).

Le diaphragme a été reséqué suivi d’une suture transversale du duodénum au vicryl 2/0 en points séparés. L’instillation de 50cc de sérum physiologique en intraluminale en aval de l’obstacle a permis d’exclure la présence des diaphragmes étagés.

En période postopératoire, le nouveau-né a été pris en charge par une équipe mixte associant, les pédiatres, les réanimateurs et les chirurgiens. Il avait bénéficié d’une antibioprophylaxie, un apport liquidien en perfusion, une analgésie et un réchauffement avec la bouillotte. L’alimentation par voie orale fait de l’allaitement maternel était permise 24 heures après l’intervention.

Son évolution était marquée par les vomissements à répétition qui avaient nécessité l’administration de l’ondansetron 0,5 mg toutes les 8 heures. Les vomissements avaient disparus avec retour efficace du transit et émission de selles 72 heures après l’intervention chirurgicale.

Les suites postopératoires ont été simples après disparition complète des vomissements. Sa sortie a été autorisée au 5ième jour et son poids à la sortie était de 2,8520kg.

## Discussion

L´atrésie duodénale, est une embryopathie qui porte sur l´intestin crânial [1] et qui entraîne une absence complète de la lumière duodénale. Des anomalies vasculaires, des anomalies de migration des cellules nerveuses et un défaut de recanalisation de la lumière duodénale pourraient être à l´origine de l´atrésie, mais la cause exacte reste inconnue. Elle peut se présenter sous trois formes, d’atrésie membraneuse sous la forme d’un diaphragme, atrésie cordonale et d’atrésie complète. Dans notre cas, il s’agit d’une atrésie sous forme d’un diaphragme.

L´incidence de l´atrésie duodénale est comprise entre 1/10 000 et 1/6 000 naissances vivantes avec un ratio garçon/fille proche de 1 [1, 4]. Dans cas présenté, il s’agit d’un garçon.

Le diagnostic d´une occlusion néonatale peut être évoqué soit en prénatal à l’échographie fœtale jusqu´à 50% des cas, par hydramnios, la dilatation de l´estomac et du duodénum proximal visible au troisième trimestre, soit en post natal précoce devant un syndrome occlusif néonatal. Pour ce cas le diagnostic a été posé en période post-natale.

Le nouveau-né a été amené en consultation à l’âge de 17 jours de vie et cela après avoir consulté plusieurs centres de la place. Passariello [5], en 2014 à Naples en Italie décrit son observation chez une fille de 14 jours, Kshirsafar [1], en Inde en 2011, décrit un cas d’un diaphragme incomplet chez un garçon de 18 mois.

Le symptôme pour lequel le nouveau-né a été amené en consultation était les vomissements bilieux de survenue précoce et persistants. Ceux-ci demeurent le maitre symptôme [6]. Son abdomen n’était pas ballonné, souple et dépressible. Ce qui est décrit dans les occlusions néonatales hautes ou occlusions à ventre plat.

La radiographie de l’abdomen sans préparation qui met en évidence une stase gastrique dans les occlusions complètes haut-situées [7], était non contributive dans notre cas. La radiographie à la gastrograhine avait montré que le transit était bloqué au niveau du troisième duodénum donnant l’image d’un dédoublement de l’estomac, pareilles images décrites par Ralahy et Shruti dans leurs cas respectifs [8, 9]. Ce qui a été confirmé par l’échographie montrant une dilatation et rétention de secrétions dans l’estomac et le duodénum et que l’obstacle était situé au niveau de la troisième portion duodénale.

Apres une incision longitudinale sur le duodénum, la technique opératoire reste l’excision de la membrane qui peut être partielle comme dans la série de Mikaelsson [10] (10 sur 16 patients) ou total comme dans le cas présenté. L’évolution post opératoire a été marquée par le vomissement pendant les 72 premières heures puis un retour du transit avec émission de selles. Signalons que son alimentation était précoce, 24heures après l’intervention et sa sortie a été autorisée au cinquième jour.

## Conclusion

L’occlusion néonatale sur diaphragme duodénal est une forme rare d’atrésie duodénale. Elle peut être complète ou incomplète. Elle doit attirer l’attention de tout médecin et devra être évoquée devant tout tableau d’occlusion intestinale haute avec vomissement précoce et persistant, ceci pouvant indiquer des investigations d’imagerie de mise au point. L’évolution dépend de la précocité du diagnostic et de la prise en charge qui est essentiellement chirurgical. Ce dernier volet, permet en même temps de confirmer le diagnostic.

## References

[1] Kshirsafar AY, Sangitsingh R, Vasisth Gauray, Nikam Yogesh P (2011). Duodenal stenosis in a child. Afr J Pediatr Surg..

[2] Chen QJ, Gao ZG, Tou JF, Qian YZ, Xiong QX (2014). Congenital duodenal obstruction in neonates: a decade's experience from one center. Word J Pediatr..

[3] Huang FC, Chuang JH, Shieh CS (1999). Congenital duodenal membrane: a ten - year review. Acta Paediatr Taiwan..

[4] Rousková B, Trachta J, Kavalcová L, Kuklová P, Kyncl M (2008). Duodenal atresia and stenosis. Cas Lek Cesk..

[5] Passariello A, Maddalena Y, Malamisura M, Rojo S, Aragione N, Iaccarino E (2014). Metabolic Alkalosis resulting from a Congenital Duodenal Diaphragm. Journal of Neonatal Surgery..

[6] Kumar Yogesh, Sharma Akshay, Sinha Shalini, Deshpande Vidyanand Pramod (2012). Duodenal webs: an experience with 18 patients. Journal of Neonatal Surgery..

[7] Moran Penco JM, Cardenal Murillo J (2007). Anomalies of intestinal rotation and fixation: consequences of late diagnosis beyond two years of age. Pediatr Surg Int..

[8] Ralahy MF, Rakotoarivony ST, Rakotomena SD, Randrianirin A (2009). Occlusion par bride de Ladd: à propos d’un cas. Revue d’Anesthésie-Réanimation et de Médecine d’Urgence..

[9] Sarkar Shruti, Apte Ashwin, Sarkar Nupur, Sarkar Dipankar, Longia Sheela (2011). Vomiting and food refusal causing failure to thrive in a 2 year old: an unusual and late manifestation of congenital duodenal web. BMJ Case Reports..

[10] Mikaelsson C, Arnbjonrnsson E, Kullendorff CM (1997). membranous duodenal stenosis. Acta Paediatr..

